# Prevalence of anxiety and depression among doctors; the unscreened and undiagnosed clientele in Lahore, Pakistan

**DOI:** 10.12669/pjms.322.8731

**Published:** 2016

**Authors:** Khaula Atif, Habib Ullah Khan, Muhammad Zia Ullah, Farrukh Saleem Shah, Abdul Latif

**Affiliations:** 1 Dr. Khaula Atif, MBBS, MCPS (Family-Medicine), DPH, DMA. Department of General Administration, Combined Military Hospital, Peshawar Cantonment, Khyber Pakhtun Khwah, Pakistan; 2 Dr. Habib Ullah Khan, MBBS, FCPS (General Surgery), FCPS (Neurosurgery). Department of Neurosurgery, Combined Military Hospital, Abbotabad Cantonment, Khyber Pakhtun Khwah, Pakistan; 3 Dr. Muhammad Zia Ullah, MBBS, MCPS (Family Medicine). Department of General Administration, Combined Military Hospital, Peshawar Cantonment, Khyber Pakhtun Khwah, Pakistan; 4 Dr. Farrukh Saleem Shah, MBBS, FCPS (Psychiatry). Department of Psychiatry, PNS Shifa Hospital, Karachi, Sindh, Pakistan; 5 Dr. Abdul Latif, MBBS, FCPS Trainee (Medicine). Department of Medicine, Khyber Teaching Hospital, Peshawar, Khyber Pakhtun Khwah, Pakistan

**Keywords:** Health Care Professional, Physician, Developing Countries, Psychological Stress

## Abstract

**Objectives:**

To analyze prevalence of anxiety and depression among doctors serving in a tertiary care hospital in Lahore, with a study of impact of relevant demographic features.

**Methods:**

A cross sectional study was conducted at Combined Military Hospital, Lahore, from February 2014 to Jan 2015. Participants were doctors serving in subject hospital for at least six months duration. Standardized Hospital Anxiety Depression Score (HADS) inventory was selected as inventory. Formal approval from hospital ethical committee and written informed consent from participants were obtained. Demographic characteristics of participants were recorded as independent variables; anxiety and depression scores being outcome variables. Data analysis was done via descriptive statistics (SPSS-20), qualitative data expressed as frequencies, percentages; quantitative as mean ± standard deviation(SD). Cross tabulation was done via chi-square, p-value < 0.05 considered as significant.

**Results:**

Out of 203 volunteers, 97(47.78%) responded. Score of anxiety was 7.04±4.470, maximum being 19, scores of depression was 4.94±3.605, maximum score being 15. Mild to moderate anxiety and depression were revealed in 33(34%) and 24(24.8%) respectively, while 7(7.2%) and 1(1.0%) had severe anxiety and depression respectively. There was strong positive relation between anxiety and depression (p<0.001). There was significant impact of service years on depression (*p*-0.011) and gender on anxiety (*p*-0.002), 9(17.31%) males and 24(53.33%) females had mild to moderate anxiety while 4(7.69%) males and 3(6.66%) females revealed severe anxiety and other variables did not reveal significant impact on HADS scores.

**Conclusion:**

Doctors showed high grades of anxiety and depression. They must be promptly screened and managed at all medical institutions.

## INTRODUCTION

In today’s world, almost everyone is familiar with the terms “Anxiety” and “Depression”. No part of the world is free from anxiety and depression.^1^ Yet as an iceberg phenomenon, these diseases are under-estimated, under-diagnosed and under-treated. Adequate mental health is basic necessity of every human being as a prerequisite to spend a good life. Anxiety and depression cast negative impacts on personal and professional life.^2^ But majority of people, even the health care professionals, are not ready to accept screening or treatment for health care problems.

Formal assessment of mental health issues including anxiety and depression is not a commonly practiced phenomenon. Literature reveals that developing countries are bearing almost two third of total psychiatric patients in the world, and the situation is expected to worsen.^3^

Doctors by the virtue of their job are at enhanced risk of carrying mental health challenges which may cause or exacerbate anxiety and depression. Mental health issues of doctors are mostly over-looked not only by public but even by the doctors themselves. Although appreciable work has been carried out in developed countries like US and Canada to evaluate psychological status of physicians, yet developing countries considerably lag behind.^4^

In Pakistan proper studies regarding anxiety and depression are scanty in general public as well as in doctors. Some literature revealed that 25% to 30% of population in Karachi was suffering from anxiety and depression.^5^ Another study documented a high prevalence of anxiety and depression among family practitioners in Karachi.^2^ It is documented in some literature that risk of harboring depression and suicidal ideation was twice in physicians as compared to general population.^6^ But effective screening of doctors is not comparable to such data.

This study was anticipated to reveal yielding results in analyzing prevalence of anxiety and depression among doctors serving in a tertiary care hospital in Lahore, with considerable analysis of contributory factors. It expected to gain fruitful guidelines for future strategies. Hospital Anxiety and Depression Scale (HADS) was selected as a hospital inventory as its scores are a useful guideline to plan lifestyle modifications and apt management for anxiety and depression.^7^

**Fig.1:**
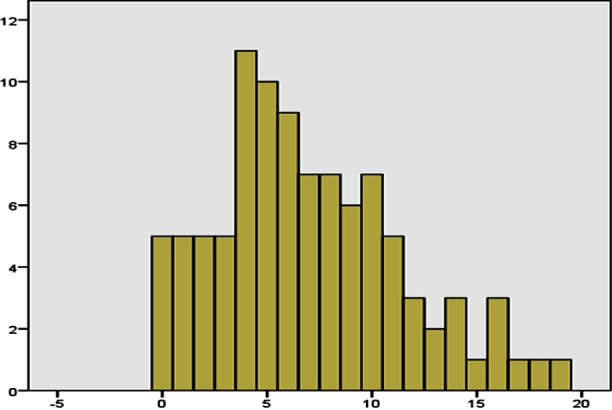
Frequency of anxiety in study Participants (n-97).

## METHODS

A cross sectional study was conducted at Combined Military Hospital, Lahore, from February 2014 to Jan 2015. Participants were doctors serving in subject hospital for at least six months duration. Doctors serving since lesser duration, already screened for, diagnosed or under treatment for any mental health issue, with history of recent stress or past mental health ailment, and those with co-morbid conditions (e.g diabetes, hypertension, tuberculosis, and chronic pain in any body part) were excluded from the study. Sampling was done by non-probability purposive technique. Standardized Hospital Anxiety Depression Scale (HADS) was selected as screening instrument. Subjects were doctors serving in tertiary care hospital for at least six months’ duration, both genders from different departments. Formal approval was taken from the ethical committee of the hospital and written informed consent from the participants. Prior estimation of non-response rate was 50%, required sample size was 96 representing a population of 20000. Therefore, 220 demographic forms and HADS-inventory were distributed to volunteers. Data analysis was done via descriptive analysis (SPSS-20), qualitative data expressed as frequencies and percentages; quantitative as mean±standard deviation(SD). Main outcome variables, anxiety and depression, were cross-tabulated with independent variables (age, marital status, number of children, education, service years, nature of job, current employment, working hours per week, additional duties, income per month in PKR, and socioeconomic class).

**Fig.2:**
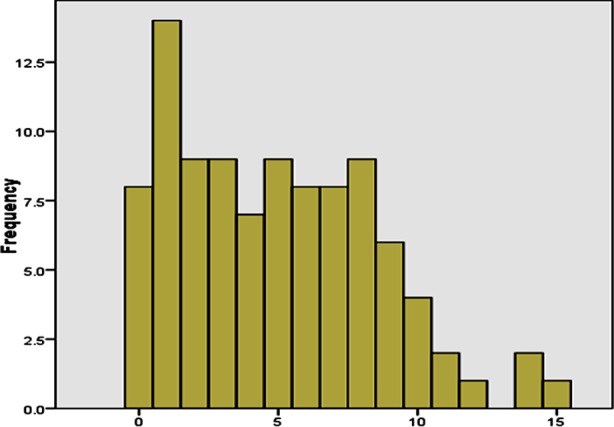
Frequency of depression in study participants (n-97).

## RESULTS

Out of 220 volunteer doctors, respondents were 47.78%(n-97). Mean age was 33.47±9.454 years. Males were 52(53.6%) and females 45(46.4%). Frequencies of other demographic characteristics are shown in Table-I. The scores of anxiety and depression are highlighted in Table-II. There was a strong positive relation between anxiety and depression scores (*p*<0.001). Impact of gender on anxiety scores was significant (*p*-0.002), 9(17.31%) males and 24 (53.33%) females had mild to moderate anxiety while 4(7.69%) males and 3(6.66%) females had severe anxiety.

**Table-I:** Demographic Characteristics of Study Participants (Frequency/Percentage) (n-97).

		*Age Group (Years)*		
22-29 Yrs (44/45.4%)	30-39 Yrs (26/26.8%)	40-49 Yrs (16/16.5%)	50-59 Yrs (10/10.3%)	≥60 Yrs (1/1.0%)

		*Marital Status*		
Single (33/34.0%)	Once Married (64/66.0%)	Divorced (0/0%)	Widowed (0/0%)	≥1Marriage (0/0%)

		*Number Of Children*		
Nil (48/48.5%)	1 (9/9.3%)	2 (11/11.3%)	3 (21/21.6%)	≥4 (8/9.3%)

		*Education*		
MBBS (61/62.9%)	MCPS And/Or MPH (4/4.1%)	Msc And/Or MPH (5/5.2%)	Single FCPS Or Equivalent (21/21.6%)	≥1 FCPS Or Equivalent (6/6.2%)

		*Service Years*		
<4 Yrs (46/47.4%)	5-9 Yrs (17/17.5%)	10-14 Yrs (7/7.2%)	15-19 Yrs (7/7.2%)	≥20 Yrs (19/19.6%)

		*Nature Of Job*		
HO^1^ (29/29.9%)	MO^2^ Administration (3/3.1%)	MO^2^ Clinical (17/17.5%)	Trainee (23/23.7%)	Consultant (25/25.8%)

		*Current Employment*		
Army Doctor (51/52.6%)	Retired From Army & Re-Employed (5/5.2%)	Civilian Doctor (41/42.2%)	--	--

		*Working Hours per Week*		
35 Hrs (6/6.2%)	36-95 Hrs (23/23.7%)	96-125 Hrs (13/13.4%)	126-175 Hrs (37/37.1%)	175-245 Hrs (19/19.6%)

		*Additional Duties*		
Nil (7/7.2%)	Resident MO^2^ (20/20.6%)	Resident (45/46.4%)	On Call From Home (16/16.5%)	Resident MO^2^+ On Call From Home (9/9.3%)

		*Income per month (Thousands PKR)*		
Nil (15/15.%)	15-49 (31/32%)	50-74 (21/21.6%)	75-99 (6/6.2%)	≥100 (24/24.7%)

		*Socio Economic Class*		
Low (2/2.1%)	Low-Middle (4/4.1%)	Middle-Middle (48/49.5%)	Upper-Middle (33/34.0%)	Upper Class (8/8.2%)

**Table-II:** Scores of Anxiety and Depression in Study Participants as per HADS (n-97).

S/No	Category	Grade	Anxiety	

			Frequency	Percent
1	Anxiety	Mild	20	20.6
		Moderate	13	13.4
		Severe	7	7.2
2	Depression	Mild	19	19.6
		Moderate	5	5.1
		Severe	1	1.0

There was no significant impact of marital status (*p*-0.157), number of children (*p*-0.505), age group (*p*-0.337), education (*p*-0.645), service years (*p*- 0.678), current employment (*p*-0.297), nature of job (*p*-0.868), working hours per week (*p*-0.338), additional duties (*p*-0.444), income per month in PKR (*p*-0.339), and socioeconomic class (*p*-0.672) on anxiety scores.

Regarding depression, doctors with lesser service had higher scores (*p*-0.011); 11.46% and 8.3% had less than 4 years and 5-9 years service respectively. The impact of gender (*p*-0.332), marital status (*p*-0.555), number of children (*p*-0.173), age group (*p*-0.075), education (*p*- 0.402), current employment (*p*-0.981), nature of job (*p*-0.423), working hours per week (*p*-0.209), additional duties (*p*-0.359), income per month in PKR (*p*-0.291), and socioeconomic class (*p*-0.555) on depression scores was not significant.

## DISCUSSION

Considerable number of people does comment at some time of their lives to have anxiety and/or depression. However, they would mostly be reluctant tor undergo formal screening or treatment. Even the patients with an insight of anxiety and depression often evade management. The mental health disorders are denied throughout the world due to fear of stigmatization.

Anxiety is defined as the state of anguish, fright or overwhelming worry^8^, which may lead to unpleasant state, undesirable psychological and physical symptoms.^9^ The low mood and repugnance to participate in daily activities is called as depression.^10^ Any anxious or depressed individual is prone to have poor mental/physical health and chances to develop personality disorders.^2^ Doctors are no exception. A psychologically unwell doctor cannot render his services compatible to his comrades who are mentally well. Doctors with anxiety or depression tend to depict poor work efficacy.^2^ The prevalence of mental disorders including anxiety/depression, substance abuse, and suicide have never been adequately studied among doctors.^6^

This study aimed to screen doctors serving at a tertiary care hospital at Lahore for anxiety and depression, with assessment of severity of disorder and allied contributory factors. HADS was selected as a hospital inventory which is an authentic, reliable and comprehensive assessment tool for anxiety or depression.^11^ It was developed by Zigmond and Snaith.^12^

In this study, 34% and 24.8% doctors had mild to moderate anxiety and depression respectively, while 7.2% and 1.0% had severe anxiety and depression respectively. None of them stated to have undergone formal screening or management yet. Enough data is not available about the anxiety and depression among doctors in Pakistan and its individual cities, yet some literature shows that 39% family practitioners in Karachi had anxiety and depression.^2^ There was a strong correlation between anxiety and depression; more anxious were more depressed and vice versa. Same is documented in other literature as well.^4^

In this study, females (59.99%) were significantly more prone to have anxiety than males (25.0%), while there was no significant impact of gender on depression scores. Literature revealed that females are more at risk to develop anxiety and depression,^4^,^13^ suicidal ideation and psychological distress^14^, six times higher risk than males doctors as per study conducted at Karachi.^2^

In this study depression was more pronounced in doctors with lesser service years, while service years had no significant impact on anxiety scores. In United Kingdom, senior medical staff had higher levels of psychological stress and anxiety.^15^ In another study, 50% of junior doctors suffered from emotional disturbance.^16^

In this study, no significant impact was revealed by marital status and additional working hours on HADS scores of the respondents. Researchers documented varying results. “Medical marriages” are documented in some studies to be unhappy ones, as physicians remain so committed in their profession that they often give precedence to professional obligations over personal happiness and leisure.^6^

Factors leading to increased rates of anxiety and depression amongst doctors include extensive workload, extended duty hours, over demanding patients, scanty resources, ethical and legal issues and traumatic or critical decision making.^2^ In Karachi, there was high association between anxiety and depression and working hours more than 48 hours per week.^2^ Another study revealed that doctors with working hours more than 46 per week were more at risk to develop psychological challenges.^14^ In Turkey physicians with low income were prone to have more anxiety.^4^

The basic aim to carry out this study was to reach productive conclusion and forthcoming tactics to attenuate the existing enigma of anxiety and depression among doctors, with a hope to develop measures which may help to decrease this incidence in upcoming days. Prevention should be employed as best cure to eliminate anxiety and depression in doctors.^2^

### Limitations

Generalization of the study cannot be warranted as all doctors of Lahore could not be screened. Response rate was low, thus selection bias was inevitable. Respondents could be more health conscious, having extra interest in medical research, could spare time to participate or they may be less afraid of stigmatization of having mental health disorder. There is a high probability of interview bias as at least some of respondents may not be willing to declare their actual mental health state.

Nevertheless, this work is unique as no such research has been conducted in doctors serving in tertiary care hospital in Lahore. The study rendered substantial data about unexpectedly high prevalence of anxiety and depression among participants. Further research and analysis may yield more prolific results, helping us to formulate guidelines to screen anxiety and depression in doctors, and deal with suffers more aptly and at early stages. Healthy doctors can warranty explicit health care delivery.

## CONCLUSION

Doctors are not immune to anxiety and depression. They should be aptly screened for anxiety and depression at regular intervals in life to avoid deterioration of their health, and to attenuate chances of patient neglect.

## References

[1] Institute of Medicine (2001). Neurological, psychiatric, and developmental disorders: meeting the challenge in the developing world.

[2] Khuwaja AK, Qureshi R (2004). Prevalence and Factors associated with Anxiety and Depression among Family Practitioners in Karachi, Pakistan. J Pak Med Assoc.

[3] Saraceno B (2001). A landmark year for world mental health 2001. J Coll Physicians Surg Pak.

[4] Erdur B, Ergin A, Turkcuer I, Parlak I, Ergin N, Boz B (2006). A study of depression and anxiety among doctors working in emergency units in Denizli, Turkey. Emerg Med J.

[5] Ali BS, Rahbar MH, Naeem S, Tareen AL, Gul A, Samad L (2002). Prevalence of and factors associated with anxiety and depression among women in a lower middle class semi-urban community of Karachi, Pakistan. J Pak Med Assoc.

[6] Miller MN, Mcgowen KR, James H (2000). The Painful Truth: Physicians Are Not Invincible. South Med J.

[7] Hansen CH, Walker J, Thekkumpurath P, Kleiboer A, Beale C, Sawhney A (2013). Screening medical patients for distress and depression: does measurement in the clinic prior to the consultation overestimate distress measured at home. Psychol Med.

[8] Iacovou S (2011). What is the Difference Between Existential Anxiety and so Called Neurotic Anxiety?: ‘The sine qua non of true vitality’: An Examination of the Difference Between Existential Anxiety and Neurotic Anxiety. Existential Analysis.

[9] Seligman MEP, Walker EF, Rosenhan DL Abnormal psychology.

[10] Salmans S (1997). Depression: Questions You Have – Answers You Need.

[11] Bjelland I, Dahl AA, Haug TT, Neckelmann D (2002). The validity of the Hospital Anxiety and Depression Scale. An updated literature review. J Psychosomatic Res.

[12] Zigmond AS, Snaith RP (1983). The hospital anxiety and depression scale. Acta Psychiatrica Scandinavica.

[13] Helbig-Lang S, Lang T, Petermann F, Hoyer J (2012). Anticipatory Anxiety as a Function of Panic Attacks and Panic-Related Self-Efficacy: An Ambulatory Assessment Study in Panic Disorder. Behavioural Cognitive Psychotherapy.

[14] Harrison D Doctors more likely to get depressed. The Age – National Newspaper.

[15] Caplan RP (1994). Stress, anxiety and depression in hospital consultants, general practitioners and senior health service managers. BMJ.

[16] Firth-Cozens J (1987). Emotional distress in junior house officers. Br Med J (Clin Res Ed).

